# KLX ameliorates liver cancer progression by mediating ZBP1 transcription and ubiquitination and increasing ZBP1-induced PANoptosis

**DOI:** 10.1038/s41401-025-01528-4

**Published:** 2025-03-27

**Authors:** Zhuo Wang, Yang Yang, Fang-ting Yao, Feng Zhang, Ke-ying Lin, Hong-tao Diao, Qiao-yue Zhao, Xue Kong, Wei Si, Ya-ting Xie, Jing-lun Song, Ling-hua Zeng, Chun-lei Wang, Yu-ting Xiong, Kun-kun Zou, Xiao-man Wang, Xin-yue Zhang, Han Wu, Wei-tao Jiang, Yu Bian, Bao-feng Yang

**Affiliations:** 1 College of Traditional Chinese Medicine and Beijing Research Institute of Chinese Medicine, Beijing University of Chinese Medicine, Beijing, 100029 China; 2 Department of Pharmacology (National Key Laboratory of Frigid Zone Cardiovascular Diseases, the State-Province Key Laboratories of Biomedicine-Pharmaceutics of China, Key Laboratory of Cardiovascular Research, Ministry of Education), College of Pharmacy, Harbin Medical University, Harbin, 150081 China

**Keywords:** liver cancer, anthraquinone, Kanglexin, PANoptosis, ZBP1, HOXD10

## Abstract

Liver cancer is a highly aggressive malignancy with poor survival rates. Current treatments, including liver transplantation, immunotherapy, and gene therapy, are often limited by late-stage diagnosis and significant side effects, highlighting the urgent need for novel therapeutic agents. In this study, we evaluated the therapeutic potential of Kanglexin (KLX), a novel anthraquinone derivative, in the treatment of liver cancer. In vitro, KLX inhibited the proliferation and migration of HepG2 and Hep3B cells in a dose-dependent manner. Mechanistically, KLX upregulated Z-DNA binding protein 1 (ZBP1) expression, inducing PANoptosis by directly binding to ZBP1, altering its conformation, and reducing its affinity for the E3 ubiquitin ligase ring finger protein 180 (RNF180). This interaction decreased ZBP1 ubiquitination, thereby increasing its stability. Additionally, KLX upregulated the expression of the transcription factor homeobox D10 (HOXD10), which further increased ZBP1 expression. Elevated ZBP1 levels significantly suppressed liver cancer cell proliferation and migration, whereas the inhibitory effects of KLX were reversed upon ZBP1 knockdown. In a xenograft model, KLX significantly inhibited tumor growth with a lower toxicity than oxaliplatin (OXA). In conclusion, KLX promoted PANoptosis in liver cancer cells by upregulating ZBP1 and preventing its degradation, thereby inhibiting liver cancer progression and migration. These findings suggest that KLX is a promising therapeutic agent for liver cancer.

## Introduction

Liver cancer, which primarily originates in the liver or intrahepatic bile ducts [1], is a highly prevalent and aggressive disease with poor survival rates, and poses a significant global health challenge [2]. Projections indicate that by 2025, liver cancer will affect more than a million individuals [1]. Although early treatments such as resection, transarterial chemoembolization (TACE), and liver transplantation are effective, they are often limited by late diagnosis and procedural complexities [3]. As a result, pharmacological intervention remains necessary. Sorafenib, a multikinase inhibitor, is the primary targeted therapy for liver cancer and acts on pathways such as the VEGFR, PDGFR, and MAPK pathways [4]. Immune checkpoint inhibitors, such as nivolumab and pembrolizumab, also play key roles in liver cancer treatment by inhibiting PD-1/PD-L1 [5]. Despite initially high response and disease control rates, these agents have not significantly improved overall survival in final clinical trials [6]. Consequently, there is an urgent need for the development of novel therapeutic agents for liver cancer.

Programmed cell death (PCD) involves evolutionarily conserved signaling pathways critical for the development of anticancer therapies [7]. The emerging concept of PANoptosis highlights the interplay and coordination among three distinct PCD pathways: pyroptosis, apoptosis, and necroptosis [8]. Research has shown that PANoptosis can effectively inhibit cancer cell proliferation and combat drug resistance, offering new avenues for targeted cancer therapy [9]. Studies have demonstrated that the loss of NFS1, in combination with oxaliplatin, increases ROS levels in colorectal cancer cells, thereby inducing PANoptosis [10]. Similarly, the combined use of metformin and doxorubicin had the same effect [11]. PANoptosis is mediated by multiprotein complexes known as PANoptosomes, which include Z-DNA binding protein 1 (ZBP1), absent in melanoma 2 (AIM2), receptor-interacting protein kinase 1 (RIPK1), and NOD-like receptor family pyrin domain containing 12 (NLRP12) [12]. These complexes integrate components from various PCD pathways [13]. ZBP1, in particular, plays a pivotal role in multiple cancers. Previous studies have shown that ZBP1 knockdown reduces the efficacy of small molecules against liver cancer [14]. However, the specific effects of ZBP1 on liver cancer cell proliferation and migration remain unexplored.

Our group has developed a novel anthraquinone derivative, 4,5-dihydroxy-7-methyl-9,10-anthraquinone-2-ethyl succinate, named Kanglexin (KLX), through chemical modification of the anthracene hydroxyl group of rhein. Previous studies have demonstrated that KLX protects the heart, prevents myocardial ischemic injury [15], delays myocardial aging [16], and reduces myocardial fibrosis [17]. However, its potential inhibitory effects on liver cancer have not yet been explored.

In this study, we found that KLX promoted PANoptosis in liver cancer cells via ZBP1, effectively inhibiting cancer cell proliferation and migration. We also discovered that KLX bound to ZBP1, altering its spatial conformation, inhibiting its interaction with RNF180, and preventing its ubiquitination, thereby increasing its protein stability. Additionally, we identified HOXD10 as a transcription factor that promoted ZBP1 transcription, which is regulated by KLX. Overall, our findings suggested that KLX is a promising, low-toxicity therapeutic agent for liver cancer, which offers a new strategy for clinical treatment.

## Materials and methods

### Cell lines and culture conditions

The human hepatoblastoma cell line HepG2 and hepatocellular carcinoma Hep3B [18] were cultured in Dulbecco’s modified Eagle’s medium (DMEM) (Biological Industries, Israel) supplemented with 10% fetal bovine serum (FBS) and 1% penicillin-streptomycin solution (Beijing Biyun Tian Biological Technology Co., Ltd., China). The cells were incubated at 37 °C in a humidified atmosphere containing 5% CO_2_.

### Xenograft tumorigenesis model

Six-week-old female nude mice (*Nu*/*Nu* strain) were obtained from Beijing Vital River Laboratory Animal Technology Limited Company (Beijing, China) and randomly assigned to five groups. A total of 1 × 10^6^ HepG2 cells were subcutaneously injected into each mouse, and the tumor volume was measured every 2 d. After 16 days of drug treatment, the mice were euthanized, and the xenograft tumors were collected. Tumor volumes were determined with the following standard formula: length × width^2^/2. All animal experiments were carried out with the endorsement of the Institutional Animal Care and Use Committee of Harbin Medical University (approval number: IRB3014822) and adhered to the principles outlined in the NIH Guide for the Care and Use of Laboratory Animals.

### Cell Counting Kit-8 assay

The Cell Counting Kit-8 (CCK-8) assay was used to evaluate cell viability. HepG2 and Hep3B cells were seeded at a density of 5 × 10^3^ cells per well in a 96-well plate. Following drug, plasmid or siRNA treatment, each well of the 96-well plate was supplemented with 10 μL of CCK-8 reagent and 100 μL of DMEM medium. The plate was then incubated for 1 h. Subsequently, the absorbance values were measured at 450 nm using a Power Wave HT microplate spectrophotometer (Bio Tek, USA). The reagent Z-VAD-FMK (#HY-16658B), necrostatin-1 (#HY-15760) and disulfiram (#HY-B0240) were purchased from MedChemExpress and used at concentrations of 20 µM, 10 µM and 1 µM, respectively.

### Colony formation assay

The cells were seeded into 6-well plates at a density of 500 cells per well. The cells were cultured at 37 °C, 5% CO_2_ incubator for 14 days to allow colony formation. The cells were subsequently fixed with 4% formaldehyde for 30 min, washed twice with PBS, and stained with 0.1% crystal violet for 20 min after air drying. Following another two washes with PBS, the colonies were photographed and counted.

### 5-Ethynyl-2’-deoxyuridine (EdU) staining assay

A Cell-Light^TM^ EdU Apollo In Vitro Kit (RiboBio, Guangzhou, China) was used to assess cell proliferation. The cells were seeded in 24-well plates (NEST, Hong Kong, China) at a density of 2 × 10^4^ cells/well. After treatment, the cells were incubated with 50 μM EdU at 37 °C for 120 min. Following permeabilization with 0.5% Triton X-100, the Apollo staining solution was added to the cell culture medium and incubated in the dark for 30 min. Finally, the cells were incubated with 20 μg/mL Hoechst 33342 for 30 min. The EdU index (%) was calculated as the average ratio of the number of EdU-positive cells to the total number of cells with a confocal laser scanning microscope.

### Flow cytometry

An Annexin V-AbFluorTM 488/PI Apoptosis Detection Kit (Abbkine, Wuhan, China) was used to assess apoptosis following the manufacturer’s instructions. The cells were rinsed with precooled PBS twice and then centrifuged at 1500 rpm for 5 min. The cells were resuspended in 400 μL of 1× binding buffer, and 5 μL of FITC-labeled Annexin V and 2 μL of propidium iodide (PI) were added to the cell suspension for staining, which was carried out for 15 min. Data analysis was performed with CytExpert software.

### Cellular thermal shift assay

The cellular thermal shift assay (CETSA) was conducted following previously described methods [19]. The cells were treated with either DMSO or KLX for 1 h. Subsequently, the cells were lysed in 100 µL RIPA buffer, and the lysate was divided into equal-volume aliquots. Each aliquot was subjected to heat treatment at a specified temperature for 5 min. After heating, protein immunoblotting was performed with established protocols as previously described.

### Western blot

Proteins were extracted from cells with RIPA buffer. Concentrations were determined with a BCA assay (Beyotime Institute of Biotechnology, China). Equal amounts of protein were separated by SDS-PAGE and transferred onto PVDF membranes. Primary antibodies against ZBP1 (1:1000; #A13899, ABclonal, China), ZBP1 (1:1000; #13285-1-AP, Proteintech, China), AIM2 (1:1000; #A3356, ABclonal, China), RIPK1 (1:1000; #A7414, ABclonal, China), Bcl-2 (1:1000; #A19693, ABclonal, China), Bax (1:1000; #60267-1, Proteintech, China), NLRP3 (1:1000; #A12694, ABclonal, China), N-GSDMD (1:1000; #AF4012-100, Affinity, USA), RIPK3 (1:1000; #17563-1-AP, Proteintech, China), p-RIPK3 (1:500; #AP1260, ABclonal, China), MLKL (1:1000; #A19685, ABclonal, China), p-MLKL (1:1000; #AP0949, ABclonal, China), IL-1β (1:500; #WL02257, Wanleibio, China), Caspase-8 p18 (1:1000; #WL00659, Wanleibio, China), HOXD10 (1:500; #TA800777S, OriGene, USA), RNF180 (1:1000; #orb541388, Biorbyt, UK), ubiquitin (1:1000; #10201-2-AP, Proteintech), and GAPDH (1:1000; #TA-08, ZSGBbio, China) were incubated overnight at 4 °C followed by secondary antibodies (1:10000; LI-COR Bioscience, Lincoln, NE, USA) for 50 min. Blots were scanned with an infrared fluorescence imaging detector (LI-COR Odyssey).

### DARTS

HepG2 cells were treated with KLX for 1 h, followed by three washes with the PBS. The cells were lysed with lysis buffer containing protease inhibitors (Roche, Switzerland) and collected after treatment on ice. The supernatant was collected by centrifugation at 12000 rpm for 10 min at 4 °C. After TNC (500 µM Tris-HCl, 500 mM NaCl, 100 mM CaCl_2_, pH 8.0) was added at a 1:10 ratio, the protein concentration was determined with a BCA protein kit (Beyotime Institute of Biotechnology, Shanghai, China), and the supernatant was divided into 3 portions. Different proportions of protease were added as needed. After heating, Western blotting was performed as described previously.

### Coimmunoprecipitation (Co-IP)

The cells were lysed in ice-cold lysis buffer (20 mM Tris-HCl pH 7.5, 150 mM NaCl, 1% NP-40, 1 mM EDTA, 1 mM PMSF, and protease inhibitor). The lysates were cleared by centrifugation at 12,000 × *g* for 15 min at 4 °C. The supernatant was incubated with 2 µg of primary antibody against ZBP1 (1:1000; #A13899, ABclonal, China) overnight at 4 °C with gentle agitation. Protein A/G agarose beads were then added and the mixture was incubated for an additional 2 h. Beads were washed five times with lysis buffer, and bound proteins were eluted by boiling in SDS sample buffer. The eluates were analyzed by SDS-PAGE and Western blotting with specific antibodies.

### Transwell assay

HepG2 and Hep3B cells were seeded at a density of 5 × 10^3^ cells per well in 24-well Transwell chambers. After 8 h, the cells were treated and incubated. After treatment, 24-well plates were filled with paraformaldehyde fixative (Biosharp, Beijing, China), and the chambers were immersed in the fixative for 30 min. Then the chambers were washed with PBS. Clean wells of a 24-well plate were then filled with crystal violet staining solution (Biosharp, Beijing, China), and the chambers were placed in the staining solution for 30 min. Cell migration was captured by imaging software, and the average number of migrated cells was calculated for quantitative analysis.

### RNA isolation and quantitative real-time PCR

Total RNA was extracted from HepG2 cells with TRIzol reagent (Invitrogen, Karlsbad, CA, USA) according to the manufacturer’s instructions. A reverse transcription kit (Toyobo, Osaka, Japan) was used to reverse transcribe 1 μg of RNA into complementary DNA (cDNA) at 37 °C for 15 min, 98 °C for 5 min, and 4 °C indefinitely. Real-time quantitative PCR was performed on a 7500 FAST Real-Time PCR System (ABI, Waltham, MA, USA) with SYBR Green (Toyobo). GAPDH was used as an internal reference to quantify messenger RNA (mRNA) levels. mRNA expression was analyzed according to the 2^−ΔΔCt^ method.

ZBP1 (forward primer: 5ʹ-AACATGCAGCTACAATTCCAGA-3ʹ; reverse primer: 5ʹ- AGTCTCGGTTCACATCTTTTGC-3ʹ);

AIM2 (forward primer: 5ʹ-TGGCAAAACGTCTTCAGGAGG-3ʹ; reverse primer: 5ʹ- AGCTTGACTTAGTGGCTTTGG-3ʹ);

RIPK1 (forward primer: 5ʹ-GGGAAGGTGTCTCTGTGTTTC-3ʹ; reverse primer: 5ʹ- CCTCGTTGTGCTCAATGCAG-3ʹ);

GAPDH (forward primer: 5ʹ-AAGAAGGTGGTGAAGCAGGC-3ʹ; reverse primer: 5ʹ-TCCACCACCCTGTTGCTGTA-3ʹ);

18S (forward primer: 5ʹ- CCTGGATACCGCAGCTAGGA-3ʹ; reverse primer: 5ʹ- GCGGCGCAATACGAATGCCCC-3ʹ);

HOXD10 (forward primer: 5ʹ- GACATGGGGACCTATGGAATGC-3ʹ; reverse primer: 5ʹ-CGGATCTGTCCAACTGTCTACT-3ʹ).

### Wound healing assay

The cells were seeded in 6-well plates. When the cells reached 90% confluence, a sterile 2.5 μL pipette tip was used to slide straight through the cell monolayer, which was photographed under a microscope. The cells were incubated in serum-free medium, and the wounds were observed and captured. The gap lengths were measured from the photomicrographs.

### Luciferase reporter assay

Binding sites between ZBP1 and HOXD10 were identified with bioinformatics tools such as microRNA.org, starBase v2.0, and miRcode. The ZBP1 3’-UTR fragment, which includes either the predicted HOXD10 binding sites or their mutated versions, was synthesized and inserted into the pGL3-basic vector (Promega, Madison, WI, USA). Human HepG2 cells were then cotransfected with this luciferase reporter vector, both in the presence and absence of the ZBP1 mimic. The Dual Luciferase Reporter Assay System (Promega) was used to assess luciferase activity following the manufacturer’s instructions. The results were normalized against Renilla luciferase activity.

### Surface plasmon resonance (SPR)

The CM5 chip was inserted into the slot with the labeled side up, and the second channel was activated by the flow of EDC and NHS at 10 μL/min. Ligand protein was immobilized at 50 μg/mL in sodium acetate buffer onto the second channel, followed by blocking with ethanolamine. The first channel served as a reference, with buffer instead of protein. Each sample was diluted to various concentrations in a 96-well plate and allowed to flow over the chip at 30 μL/min for 150 s. The chip was regenerated with 10 mM glycine-HCl (pH 2.0) after each concentration, and the binding constants were determined using Biacore Insight software.

### Molecular docking

The crystal structure of ZBP1 was retrieved from the RCSB PDB. Molecular docking was carried out with AutoDock Vina 1.1.2, and visual inspection was performed with PyMol 2.5.4. Interaction energies were predicted through flexible ligand docking simulations within the monomeric pores.

### Molecular dynamics

The protein crystal structures of ZBP1, BMI1, and SYVN1 were downloaded from the PDB database [20]. RNF180 was obtained from the AlphaFold database, and the 3D structure of KLX was downloaded from PubChem and energy-minimized with the MMFF94 force field [21]. Protein-protein docking was performed using ZDOCK 3.0.2. KLX and ZBP1 docking was performed with AutoDock Vina 1.1.2, with receptor proteins preprocessed in PyMol and converted to PDBQT format by ADFRsuite 1.04. Energy minimization and molecular dynamics simulations were conducted with AMBER 18, followed by MMGBSA binding free energy calculations based on 90-100 ns MD trajectories.

### Statistical analysis

The data are shown as the means ± SDs. Statistical analysis was performed with Student’s *t*-test for comparing two groups and one-way ANOVA with Tukey’s *post hoc* test for multiple group comparisons. A *P* value of less than 0.05 was considered statistically significant. Statistical analysis was conducted with GraphPad Prism 9.

## Results

### KLX inhibited the proliferation and migration of HepG2 and Hep3B cells

**Fig. 1:**
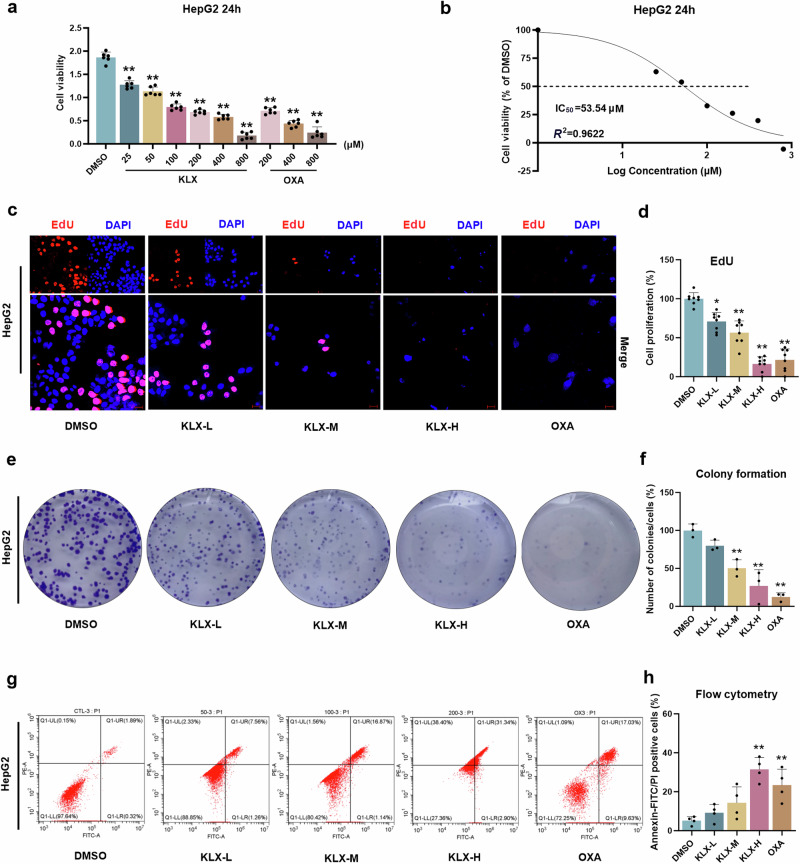
Effects of KLX on HepG2 cell proliferation. **a**, **b** CCK-8 assay results showing the viability of HepG2 cells after 24 h of treatment with various concentrations of KLX (25, 50, 100, 200, 400 or 800 μM) and OXA (200, 400 or 800 μM). The IC_50_ for HepG2 cells was determined to be 53.54 μM. *n* = 6. **c**, **d** Results from the EdU staining assay showing the effects of the KLX and OXA treatments on HepG2 cell proliferation, including the corresponding statistical analysis. *n* = 8. **e**, **f** Colony formation assay confirming the effects of the KLX and OXA treatments on HepG2 cell proliferation. *n* = 3. **g**, **h** Flow cytometry results showing the effects of KLX and OXA on the apoptosis of HepG2 cells. Early and late apoptotic cells, corresponding to the Q1-LR and Q1-UR regions in the figure, were included in the statistical analysis. *n* = 4. All the data are expressed as the means ± SDs; *P < 0.05, **P < 0.01, vs. the DMSO group.

To evaluate the effect of KLX on liver cancer cell proliferation, KLX was tested with a CCK-8 assay. OXA was used as a positive control because of its established clinical efficacy against liver cancer. The results revealed that KLX significantly reduced the viability of HepG2 and Hep3B cells, with IC_50_ values of 53.54 μM and 63.07 μM, respectively (Fig. 1a, b; Fig. S1a, b). On the basis of these findings, we administered KLX at concentrations of 50, 100, and 200 μM, which corresponded to the low (KLX-L), medium (KLX-M), and high (KLX-H) dose groups, respectively, while OXA was used at 200 μM for comparison. The results of the EdU assay demonstrated that KLX suppressed cell proliferation in a dose-dependent manner (Fig. 1c, d; Fig. S1c, d), which was further supported by the results of the colony formation assay (Fig. 1e, f; Fig. S1e, f). Additionally, flow cytometry analysis revealed that KLX significantly induced cell death (Fig. 1g, h; Fig. S1g, h). These findings indicate that KLX effectively inhibited liver cancer cell proliferation in vitro.

**Fig. 2:**
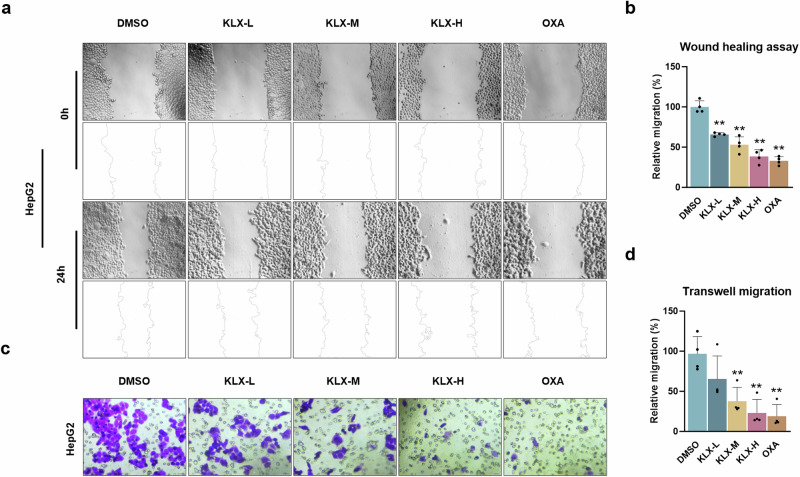
The effect of KLX on HepG2 cell migration. **a**, **b** Wound healing assay results showing HepG2 cell migration after treatment with KLX or OXA, with images taken at 0 h and 24 h. *n* = 4. **c**, **d** Transwell assays were also used to evaluate HepG2 cell migration. *n* = 4. All the data are expressed as the means ± SDs; *P < 0.05, **P < 0.01, vs. the DMSO group.

To verify whether KLX affects liver cancer cell migration, wound healing assays were performed on the HepG2 and Hep3B cell lines. The results demonstrated that KLX inhibited cell migration in a concentration-dependent manner (Fig. 2a, b; Fig. S2a, b). These findings were further corroborated by Transwell assays (Fig. 2c, d; Fig. S2c, d). Collectively, these data indicate that KLX significantly suppressed the migration of liver cancer cells.

### KLX induced PANoptosis in liver cancer cells

**Fig. 3:**
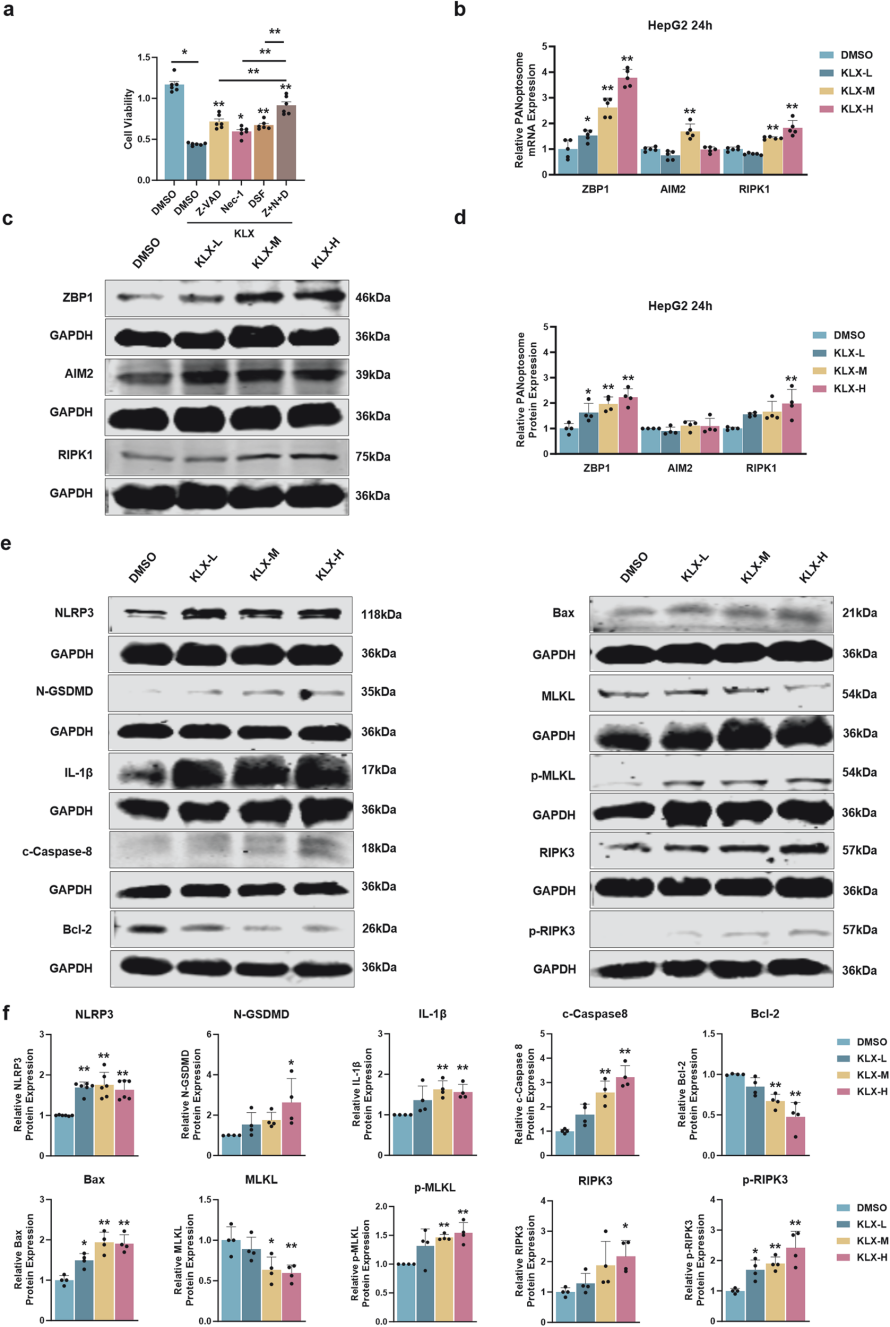
Validation of KLX-mediated effects on PANoptosis. **a** CCK-8 assay results showing that preincubation with the pancaspase inhibitor Z-VAD, the necroptosis inhibitor Nec-1, and the pyroptosis inhibitor DSF for 12 h significantly increased the inhibitory effect of KLX on cell viability. *P < 0.05, **P < 0.01. **b** qPCR was used to validate the effects of different concentrations of KLX on the mRNA levels of three PANoptosome proteins, ZBP1, AIM2, and RIPK1. *n* = 5. **c**, **d** Western blot analysis validated the effects of different concentrations of KLX on the protein levels of three PANoptosome proteins, ZBP1, AIM2, and RIPK1. *n* = 4. **e**, **f** Western blot analysis validated the effects of different concentrations of KLX on the protein levels of apoptosis-, pyroptosis- and necroptosis-related markers. *n* = 4–6. All the data are expressed as the means ± SDs; *P < 0.05, **P < 0.01, vs. the DMSO group.

Given that KLX induced cell death, we sought to elucidate the specific mechanisms underlying its cytotoxic effects. A CCK-8 assay demonstrated that treatment with the cell death inhibitors Z-VAD-FMK (Z-VAD), necrostatin-1 (Nec-1), and disulfiram (DSF) significantly reversed KLX-induced cytotoxicity. Notably, the combined application of the three inhibitors had a stronger effect than that of each inhibitor used individually, suggesting the involvement of pancaspase, pyroptosis, and necroptosis pathways (Fig. 3a). These results suggest that PANoptosis may be involved. To explore this hypothesis further, we examined the associations of KLX with the PANoptosome components ZBP1, AIM2, and RIPK1. qPCR analysis revealed that KLX significantly upregulated the mRNA expression of these components in a dose-dependent manner, with ZBP1 exhibiting the most substantial increase (Fig. 3b). These findings were also corroborated by Western blot analysis (Fig. 3c, d). Additionally, we assessed the expression of key markers associated with apoptosis, pyroptosis, and necroptosis by Western blotting (Fig. 3e, f). We found that KLX significantly upregulated the proapoptotic proteins Bax and cleaved caspase-8, while downregulating the antiapoptotic protein Bcl-2. KLX also increased the expression of the pyroptosis markers NLRP3, IL-1β, and N-GSDMD, as well as the necroptosis-related proteins p-RIPK3 and p-MLKL. Collectively, these findings indicate that KLX induced PANoptosis in liver cancer cells by upregulating ZBP1 expression at both the RNA and protein levels.

### KLX directly bound to ZBP1 and inhibited its ubiquitination

**Fig. 4:**
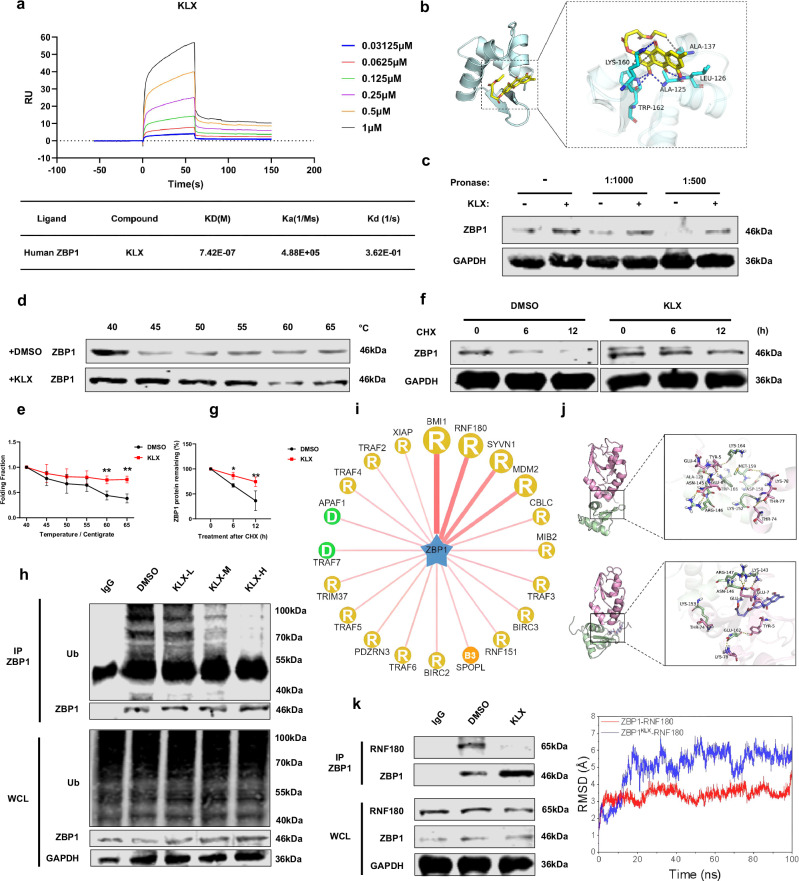
Assessment of the direct regulatory effect of KLX on the ZBP1 protein. **a** SPR experiments validated the binding affinity between KLX and ZBP1. **b** Docking prediction of the direct interaction between ZBP1 and KLX revealed that the amino acid residues LYS-160, ALA-137, LEU-126, ALA-125 and TRP-162 in ZBP1 have strong binding affinities with KLX. **c** DARTS analysis verified the effect of KLX on ZBP1 protein stability. *n* = 4. **d**, **e** CETSA was used to evaluate the degradation levels of the ZBP1 protein in the DMSO and KLX groups as the temperature increased; the associated statistical analysis of the results is shown in the graph. *n* = 5. The data are expressed as the means ± SDs; *P < 0.05, **P < 0.01, vs. the DMSO group. **f**, **g** Western blot experiments verified the degradation of the ZBP1 protein in the DMSO and KLX groups after treatment with cycloheximide (CHX) for 0, 6 or 12 h; the statistical analysis of the results is shown in the graph. *n* = 4. **h** Co-IP assays verified the effects of different concentrations of KLX on the ubiquitination of the ZBP1 protein. *n* = 3. **i** Website prediction of ubiquitin enzymes associated with ZBP1. **j** Molecular dynamics simulation was used to assess the effect of KLX on the binding affinity between ZBP1 and RNF180. The figure shows ZBP1 in green, KLX in blue, RNF180 in pink, and yellow dashed lines for hydrogen bonds. The upper image illustrates protein binding without KLX, and the lower image shows binding with KLX. **k** Co-IP assays verified the effect of KLX on the interaction between ZBP1 and RNF180. *n* = 4.

Given that KLX upregulated ZBP1 protein levels, we aimed to determine whether it directly binds to ZBP1 and influences its stability. SPR experiments revealed a dissociation constant (*K*_D_) of 7.42 × 10^-7 ^M between KLX and ZBP1, indicating a strong interaction (Fig. 4a). Molecular docking simulations further revealed a binding affinity of –6.263 kcal/mol between KLX and ZBP1 (Fig. 4b). To validate the effect of KLX on ZBP1 stability, we conducted DARTS and CETSA analyses, which confirmed that KLX inhibited ZBP1 degradation (Fig. 4c–e). Additionally, cells treated with 50 µg/mL cycloheximide for 0, 6 or 12 h presented significantly lower ZBP1 protein levels in the DMSO group than in the KLX group, further supporting the role of KLX in stabilizing ZBP1 (Fig. 4f, g). Co-IP experiments revealed that KLX significantly reduced ZBP1 ubiquitination in a dose-dependent manner, suggesting that KLX enhanced ZBP1 stability by preventing its ubiquitin-mediated degradation (Fig. 4h). To identify the ubiquitin ligases involved, we used web-based prediction tools (UbiBrowser) to rank potential ubiquitin enzymes on the basis of binding affinity (Fig. 4i). Molecular dynamics simulations indicated that KLX significantly reduced the binding energy between ZBP1 and RNF180, but not between ZBP1 and other ligases such as BMI1 or SYVN1 (Fig. 4j; Fig. S3a, b). Subsequent Co-IP experiments confirmed that KLX significantly inhibited the interaction between ZBP1 and RNF180 (Fig. 4k). These findings suggest that KLX directly bound to ZBP1, altering its conformation and thereby increasing its stability by inhibiting ubiquitination.

### KLX modulated ZBP1 transcriptional activity via HOXD10 regulation

**Fig. 5:**
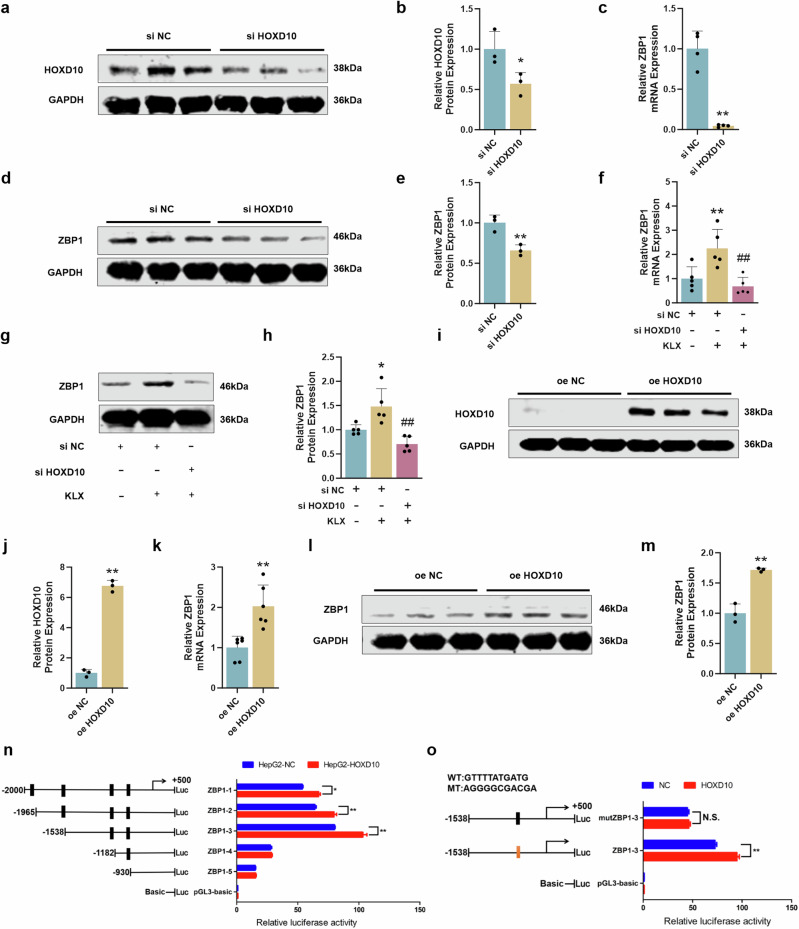
Verification of KLX-induced ZBP1 transcription via HOXD10. **a**, **b** Western blot analysis was used to assess the efficiency of HOXD10 knockdown; the statistical analysis of the results is shown in the graph. *n* = 3. The data are expressed as the means ± SDs; *P < 0.05, *vs*. the siRNA NC group. **c** qPCR analysis verified the mRNA expression level of ZBP1 after HOXD10 knockdown. *n* = 4. The data are expressed as the means ± SDs; **P < 0.01, *vs*. the siRNA NC group. **d**, **e** Western blot analysis was used to assess the protein expression of ZBP1 after HOXD10 knockdown; the statistical analysis of the results is shown in the graph. The data are expressed as the means ± SDs; **P < 0.01, vs. the siRNA NC group. *n* = 3. **f** qPCR confirmed that HOXD10 knockdown inhibited KLX activation of ZBP1 mRNA. The data are expressed as the means ± SDs; **P < 0.01, vs. the siRNA NC group, ^##^*P* < 0.01, vs. the siRNA NC + KLX group. *n* = 5. **g**, **h** Western blot analysis validated ZBP1 protein expression; the statistical analysis of the results is shown. The data are expressed as the means ± SDs; *P < 0.05, vs. the siRNA NC group, ^##^*P* < 0.01, vs. the siRNA NC + KLX group. *n* = 5. **i**, **j** Western blot analysis was used to assess the efficiency of HOXD10 overexpression, and the statistical analysis of the results is shown in the graph. *n* = 3. The data are expressed as the means ± SDs; **P < 0.01, vs. the oe NC group. **k** qPCR analysis verified the mRNA expression level of ZBP1 after HOXD10 overexpression. *n* = 6. The data are expressed as the means ± SDs; **P < 0.01, vs. the oe NC group. **l**, **m** Western blot analysis was used to assess the protein expression of ZBP1 after HOXD10 overexpression; the statistical analysis of the results is shown in the graph. *n* = 3. All the data are expressed as the means ± SDs; **P < 0.01, vs. the oe NC group. **n** Luciferase assays verified the binding of HOXD10 to the ZBP1 promoter sequence. The fluorescence intensity was assessed after binding of HOXD10 to five different fragments of the ZBP1 sequence. **o** Changes in fluorescence intensity after mutation of the binding site of ZBP1-3, which had the strongest binding affinity, to observe its interaction with HOXD10. The data are expressed as the means ± SDs; *P < 0.05, **P < 0.01, vs. the NC group.

Owing to the significant upregulation of ZBP1 mRNA induced by KLX, we investigated whether this compound stimulated ZBP1 transcription through a specific transcription factor. HOXD10 is a key transcription factor with known tumor-suppressive effects in various cancers [22, 23]. Therefore, we aimed to verify whether HOXD10 is a potential regulatory factor of ZBP1. To assess the role of HOXD10 in KLX-mediated ZBP1 regulation, we knocked down HOXD10 in HepG2 cells with small interfering RNA (siRNA) (Fig. 5a, b). Both the mRNA and protein levels of ZBP1 were significantly lower than those in the negative control (NC) group (Fig. 5c–e). Additionally, the upregulation of ZBP1 by KLX was markedly diminished upon HOXD10 knockdown (Fig. 5f–h). To further validate the role of HOXD10 in regulating ZBP1, we overexpressed HOXD10 with a plasmid construct. Western blot analysis confirmed successful overexpression (Fig. 5i, j), and both qPCR and Western blotting revealed increased ZBP1 expression upon HOXD10 upregulation (Fig. 5k–m). To confirm that ZBP1 is a direct target of HOXD10, we divided the ZBP1 promoter sequence into five segments (ZBP1-1 to ZBP1-5). Luciferase assays revealed that segments 1, 2, and 3 bound to HOXD10, with segment 3 exhibiting the highest binding activity (Fig. 5n). Mutation of the predicted binding sites in the ZBP1-3 promoter abolished the increase in promoter activity, confirming that ZBP1-3 is the binding site for HOXD10 (Fig. 5o). Moreover, qPCR and Western blot experiments confirmed that combined treatment with KLX and oe HOXD10 significantly upregulated ZBP1 mRNA and protein levels (Fig. S4a–c). To clarify whether KLX first regulates HOXD10-mediated mRNA transcription or ZBP1 protein degradation, KLX was added to the cells for 0, 6, 12 and 24 h, followed by the extraction of RNA and protein. The ZBP1 mRNA levels significantly increased after 12 h of KLX treatment, whereas noticeable changes in protein expression occurred at 24 h (Fig. S4d–f). These results suggest that KLX primarily increased the HOXD10-mediated transcription of ZBP1.

### KLX inhibited liver cancer progression via ZBP1

To assess the impact of ZBP1 on liver cancer cells, we overexpressed ZBP1 with a plasmid, and confirmed the overexpression efficiency via Western blot analysis (Fig. S5a, b). ZBP1 overexpression significantly reduced cell viability (Fig. S5c) and inhibited cell proliferation, as demonstrated by colony formation and EdU assays (Fig. S5d–g). Additionally, flow cytometry analysis revealed a marked increase in apoptosis following ZBP1 overexpression (Fig. S5h, i). Moreover, Transwell and wound healing assays indicated that ZBP1 inhibited HepG2 cell migration (Fig. S5j–m). Collectively, these findings suggest that ZBP1 overexpression inhibited both the proliferation and migration of liver cancer cells.

**Fig. 6:**
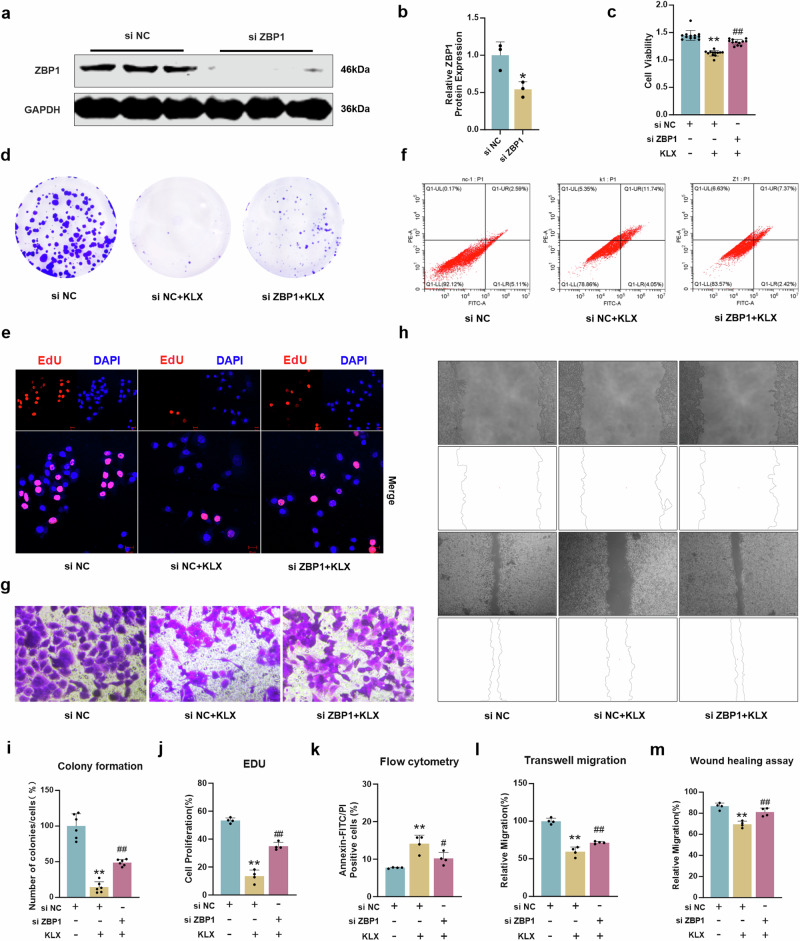
ZBP1 knockdown reversed the effect of KLX on HepG2 cells. **a**, **b** Western blot analysis verified the efficiency of ZBP1 knockdown. *n* = 3. **c** CCK-8 assays verified the reversal effect of ZBP1 knockdown on the KLX-induced decrease in cell viability. *n* = 12. **d**–**h** Colony formation (*n* = 6), EdU (*n* = 4), flow cytometry (*n* = 4), Transwell (*n* = 4) and wound healing (*n* = 4) assays verified the reversal effect of ZBP1 knockdown on the KLX-induced decrease in cell viability. **i**–**m** Statistical graph of the results of the colony formation, EdU, flow cytometry, Transwell and wound healing assays. All the data are expressed as the means ± SDs; *P < 0.05, **P < 0.01, vs. the siRNA NC group; ^#^*P* < 0.05, ^##^*P* < 0.01, vs. the siRNA NC + KLX group.

To determine whether the suppressive effects of KLX on liver cancer were mediated through ZBP1, we effectively silenced ZBP1 expression with siRNA (Fig. 6a, b). KLX significantly reduced cell viability compared with that in the NC group; however, this reduction was reversed by ZBP1 knockdown (Fig. 6c). Similar trends were observed in colony formation, EdU and flow cytometry assays (Fig. 6d–f, i–k). Additionally, ZBP1 silencing reversed cell migration, as shown by the Transwell and wound healing assays (Fig. 6g, h, l, m). These findings strongly suggest that KLX promoted liver cancer cell death and inhibited migration through the upregulation of ZBP1.

### KLX inhibited liver cancer tumor growth in a postsurgical residual tumor xenograft model

**Fig. 7:**
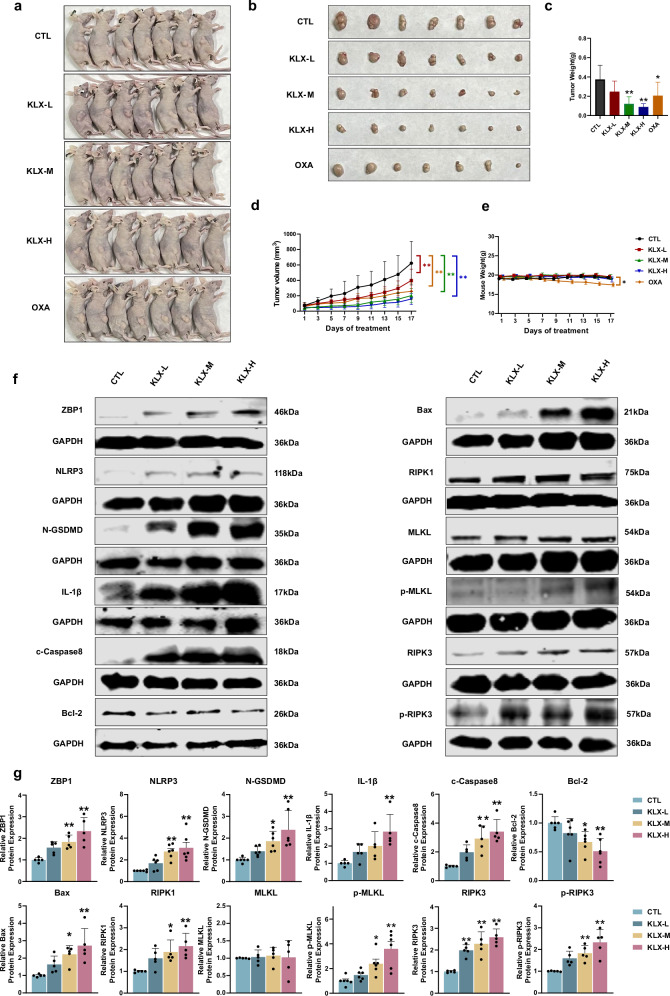
Tumor changes in nude mice after 16 d of KLX administration in a subcutaneous tumor model. **a**, **b** Images of nude mice and liver cancer tumors after 16 d. *n* = 7. **c** Tumor weight change in nude mice after 16 d. **d** Tumor volume in nude mice after 16 d. **e** Mouse weight changes after 16 d. *n* = 7. **f**, **g** Western blot experiments showing the expression of PANoptotic proteins in tumor tissues at different concentrations of KLX, along with related statistical data. *n* = 5-6. All the data are expressed as the means ± SDs; *P < 0.05, **P < 0.01, vs. the CTL group.

To investigate the effects of KLX on nude mice, HepG2 cells were used to establish a xenograft tumor model, and the mice were randomly divided into five groups: CTL, KLX-L (50 mg/kg), KLX-M (100 mg/kg), KLX-H (200 mg/kg), and the positive control OXA (3 mg/kg). KLX was administered daily via gavage, whereas OXA was given every 3 d via intragastric injection. Tumor size and body weight were monitored every other day. After 16 d of treatment, the mice were photographed, and the tumors were harvested for further analysis. As shown in Fig. 7a–d, KLX significantly inhibited tumor growth in a dose-dependent manner. Importantly, KLX at all the tested doses did not affect the body weight of the mice, whereas OXA treatment led to a significant reduction in body weight (Fig. 7e). Furthermore, the effect of KLX on PANoptosis-related proteins in tumor tissues was consistent with that observed in cells (Fig. 7f, g). These findings demonstrate that KLX effectively inhibited liver cancer growth with low toxicity in nude mice.

## Discussion

**Fig. 8:**
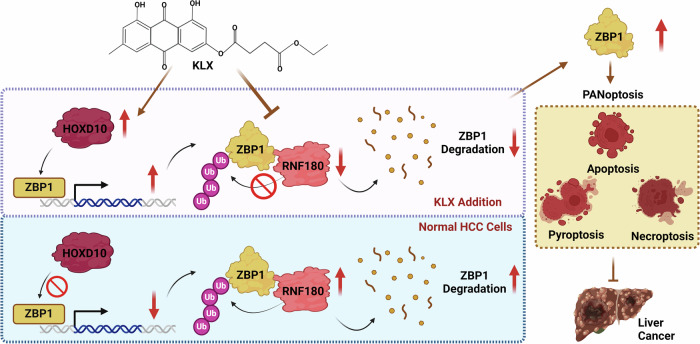
KLX modulates PANoptosis in liver cancer via ZBP1-mediated pathways. KLX increased the expression of the transcription factor HOXD10, which bound to the ZBP1 promoter and activated its transcription. KLX also inhibited the interaction between ZBP1 and the ubiquitin ligase RNF180, preventing ZBP1 protein degradation. These effects led to increased ZBP1 levels, promoted PANoptosis in liver cancer cells, and inhibited cancer progression, suggesting new therapeutic value for the use of KLX in liver cancer treatment.

Our study identified KLX as a novel compound that inhibited liver cancer progression by promoting PANoptosis in liver cancer cells. Notably, we identified for the first time the function of ZBP1 in liver cancer cells and demonstrated that KLX increased ZBP1 stability by changing its spatial conformation, which prevented its ubiquitination and subsequent degradation. Furthermore, our findings were the first to reveal HOXD10 as a previously unrecognized transcription factor that regulates ZBP1 transcription and promotes its RNA expression, which is also modulated by KLX (Fig. 8).

Liver cancer, with its heterogeneous nature and poor response to standard treatments, results in unfavorable clinical outcomes and poses a significant burden on the global health care system [4, 24]. To date, randomized clinical trials of adjuvant therapies for liver cancer have yielded largely negative results, highlighting the limitations of current treatment options [25]. Among the drugs used to treat liver cancer, oxaliplatin has not only been approved for systemic chemotherapy in liver cancer patients [26] but also serves as a primary drug for hepatic arterial infusion chemotherapy [27]. However, its clinical use is restricted by its significant toxicity and high levels of drug resistance [27]. In the study by Liu et al., a marked reduction in body weight was observed in mice treated with oxaliplatin [28], which is consistent with the findings of this study. The above experimental results indicated that KLX significantly inhibited the progression of liver cancer. In in vitro experiments, KLX at the same molar concentration as OXA had a nearly comparable inhibitory effect on liver cancer cells. In vivo experiments revealed that compared with OXA, KLX-H had a greater anticancer effect, and the toxicity in the KLX group was significantly lower than that in the OXA positive control group, with no significant effect on body weight after two weeks of continuous administration. This may be related to the reduced systemic toxicity and laxative effects of KLX through chemical modification.

KLX is a newly synthesized compound developed by our team through structural modification of a derivative extracted from emodin. KLX was shown to alleviate cardiac dysfunction and fibrosis induced by pressure overload through the noncanonical TGF-β1/ERK1/2 pathway [17]. KLX has also been shown to activate the AMPK/SREBP-2/PCSK9/LDLR signaling pathway to lower blood lipid levels and alleviate hepatic lipid accumulation [29]. Importantly, KLX can ameliorate myocardial infarction-induced cardiac damage by inhibiting the expression of the NLRP3 inflammasome, reducing the release of proinflammatory cytokines IL-1β and IL-18, and suppressing pyroptotic cell death [15]. Cardiac glycosides, well-known Na^+^/K^+^-ATPase inhibitors, exert significant cardioprotective effects by increasing myocardial contractility, reducing heart rate, and alleviating congestive heart failure [30]. Recent studies have demonstrated that, in addition to their cardioprotective properties, cardiac glycosides can inhibit tumor progression by disrupting DNA repair mechanisms in cancer cells [31]. Given that cancer cells proliferate more rapidly and are therefore more susceptible to DNA damage, cardiac glycosides exhibit enhanced cytotoxicity toward tumors. This dual action of cardiac glycosides—cardioprotection and tumor inhibition—highlights their therapeutic potential [31, 32]. Similarly, the compound KLX, as discussed in this study, not only protected the heart but also induced PANoptosis in tumor cells, which may be associated with the aforementioned mechanism. This dual functionality could also explain why high doses of KLX do not adversely affect the body weight of mice.

Under certain conditions, cells can undergo different types of PCD through interconnected pathways under certain conditions, a concept known as PANoptosis. PANoptosis involves inflammatory PCD mediated by PANoptosome complexes, which combine aspects of pyroptosis, apoptosis, and necroptosis [33]. A key component of the PANoptosome is ZBP1, initially identified as a DNA sensor, which plays a role similar to that of caspase-11 (or caspase-4/5) in LPS-induced noncanonical NLRP3 inflammasome activation [8]. Upon IAV stimulation, ZBP1 activates and triggers the assembly of the PANoptosome, which includes RIPK3, caspase-8, caspase-6, ASC, and NLRP3 [34]. ZBP1 plays crucial biological roles in various types of cancer, such as breast cancer [35] and myeloma [36]. The RIPK3 promoter is reported to be hypermethylated in HepG2 cells [37]. A previous study suggested that HSV-1-based OVs activate ZBP1 in tumor cells by increasing the level of endogenous Z-RNA. Combining *Fusobacterium nucleatum* outer membrane vesicles with HSV-1-based OVs inhibited tumor growth and increased immune checkpoint blockade efficacy [38], highlighting the importance of ZBP1 expression in the anticancer process. This study is the first to report the direct role of ZBP1 in liver cancer, highlighting its ability to promote liver cancer cell death via PANoptosis. Furthermore, network prediction analysis identified several other potential protein targets for KLX, including iNOS, XDH, Hsp90, PKCδ and MPK-9 (Swiss Target Prediction, Superpred), which warrants further investigation in future studies.

In this study, we first revealed that KLX changed the spatial conformation of the ZBP1 protein and increased its stability, reducing its degradation, which is often associated with posttranslational modifications. Ubiquitination is a conserved posttranslational modification process in which proteins are typically tagged by three types of ubiquitin enzymes: activating enzymes (E1), conjugating enzymes (E2) and ubiquitin ligases (E3), leading to protein degradation via the proteasome system [39, 40]. RING finger gene 180 (RNF180), an innovative E3 ubiquitin ligase, is comprised of a RING finger domain for ubiquitin ligase functionality and a fundamental coiled-coil domain. Mounting evidence has revealed that RNF180 is irregularly expressed in numerous human cancers, including stomach cancer, pulmonary cancer, and colon cancer [41]. In osteosarcoma, the upregulation of RNF180 augments the ubiquitination of CBX4, consequently diminishing CBX4 expression in cells [41]. In this study, we first predicted through bioinformatics analysis that RNF180 could be the ubiquitin ligase responsible for ZBP1 degradation. Molecular dynamics simulations further revealed that the introduction of KLX altered the spatial conformation of the RNF180-ZBP1 interaction, reducing the binding affinity between the two proteins. As a result, KLX directly bound to ZBP1, altering its spatial conformation to inhibit its interaction with RNF180, thereby preventing ZBP1 degradation and increasing its protein stability.

While SPR and docking experiments confirmed the direct binding of KLX to ZBP1, our previous findings revealed that KLX also significantly increased ZBP1 mRNA levels, suggesting potential regulation at the RNA level. This led us to investigate the transcription factor HOXD10 as a possible mediator. HOXD10, a member of the homeobox (HOX) superfamily, plays a crucial role in cell differentiation and morphogenesis and acts as a key transcription factor in various cancers [22]. For example, HOXD10 overexpression in colon cancer inhibits the RHOC/AKT/MAPK pathway, thereby suppressing cell proliferation, migration, and invasion [42]. In hepatocellular carcinoma, HOXD10 has been shown to inhibit proliferation by suppressing the ERK signaling pathway [43]. Our study is the first to reveal that HOXD10 can regulate ZBP1 transcription. Although ZBP1-mediated PANoptosis is known to be triggered dependently, primarily sensing IAV stimulation through its Zα domain [44], other studies have highlighted IRF1 as a crucial transcriptional regulator of ZBP1 when it is upregulated by IFN stimulation [45]. Here, we found that KLX increased HOXD10 levels, which in turn promoted ZBP1 transcription and inhibited liver cancer cell growth. However, the mechanism by which KLX regulates HOXD10 expression remains unclear. Research has suggested that microRNAs such as miR-501 [23] and miR-376b [46] can modulate HOXD10 expression, potentially influencing cancer progression. KLX may regulate HOXD10 expression by affecting the expression of specific miRNAs. Additionally, HOXD10 is known to undergo extensive methylation in various cancers [43], leading to gene silencing. It is possible that KLX inhibits HOXD10 methylation, although further experimental validation is needed to confirm these hypotheses. The specific regulatory mechanisms require further investigation. KLX has also been shown to suppress liver fibrosis and prevent atherosclerosis [47, 48], providing further evidence for the clinical application of this drug.

## Conclusions

In conclusion, our study provided compelling evidence that KLX effectively inhibited liver cancer cell proliferation and migration in vitro and suppressed tumor growth in vivo. Mechanistically, KLX was found to directly bind to ZBP1, preventing its ubiquitination and subsequent degradation, thereby increasing its protein stability. Additionally, KLX activated the transcription factor HOXD10, which in turn increased ZBP1 transcription and elevated its mRNA expression. This upregulation of the PANoptosome component ZBP1 triggered PANoptosis, ultimately promoting liver cancer cell death. Our findings establish KLX as a novel, highly effective, and low-toxicity therapeutic agent for liver cancer, suggesting a promising new strategy for clinical treatment.

## Supplementary information

Supplementary Information
