# Supplementary material for: KLX ameliorates liver cancer progression by mediating ZBP1 transcription and ubiquitination and increasing ZBP1-induced PANoptosis
Source: Acta Pharmacol Sin. 2025 Mar 27;46(8):2282–95. doi: 10.1038/s41401-025-01528-4 (PMC12274451; doi:10.1038/s41401-025-01528-4)
Supplement: Supplementary file 1 — Supplementary Information [file 41401_2025_1528_MOESM1_ESM.pdf]

Supplementary Information

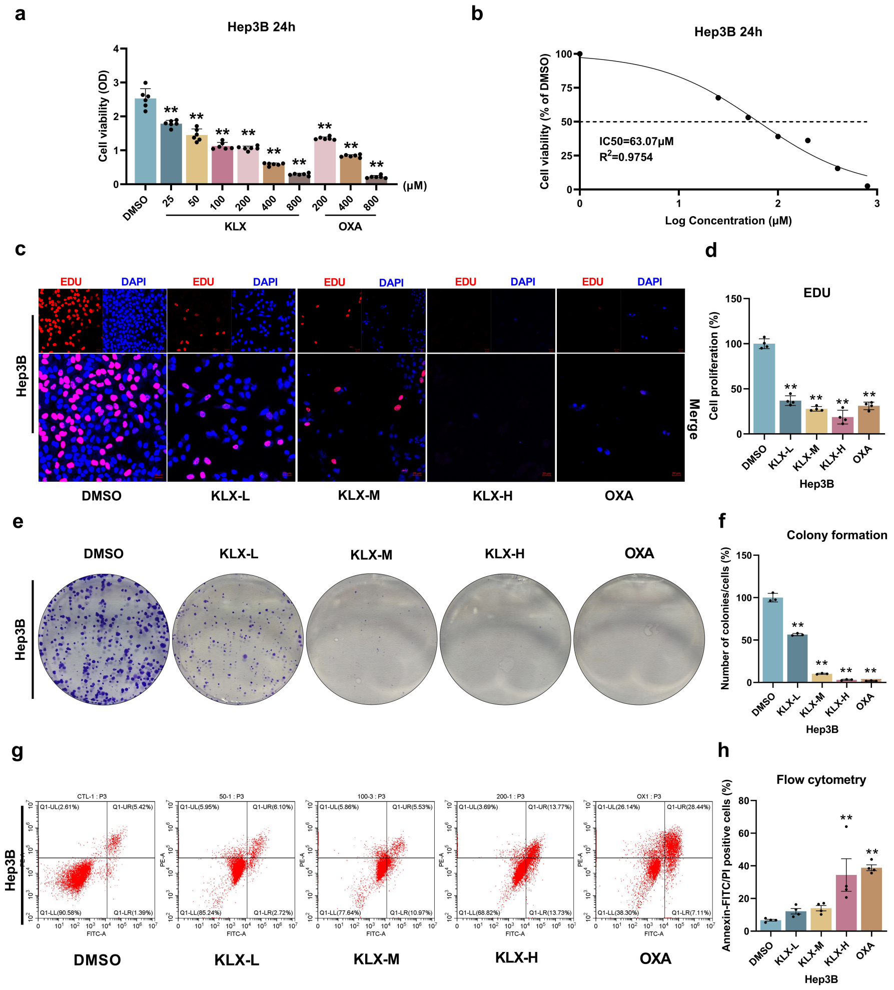

Fig. S1 Effects of KLX on Hep3B cell proliferation. **a, b** CCK-8 assay results showing the viability of Hep3B cells after 24 h of treatment with various concentrations of KLX (25, 50, 100, 200, 400 or 800 μM) and OXA (200, 400 or 800 μM). The IC50 for Hep3B cells was determined to be 63.07 μM. *n*=6. **c, d** Results from the EdU staining assay showing the effects of the KLX and OXA treatments on Hep3B cell proliferation, including the corresponding statistical analysis. *n*=4. **e, f** Colony formation assay confirming the effects of the KLX and OXA treatments on Hep3B cell proliferation. *n*=3. **g, h** Flow cytometry results showing the effects of KLX and OXA on the apoptosis of Hep3B cells. Early and late apoptotic cells, corresponding to the Q1-LR and Q1-UR regions in the figure, were included in the statistical analysis. *n*=4. All the data are expressed as the means ± SDs; **P* < 0.05, ***P* < 0.01, *vs*. the DMSO group.

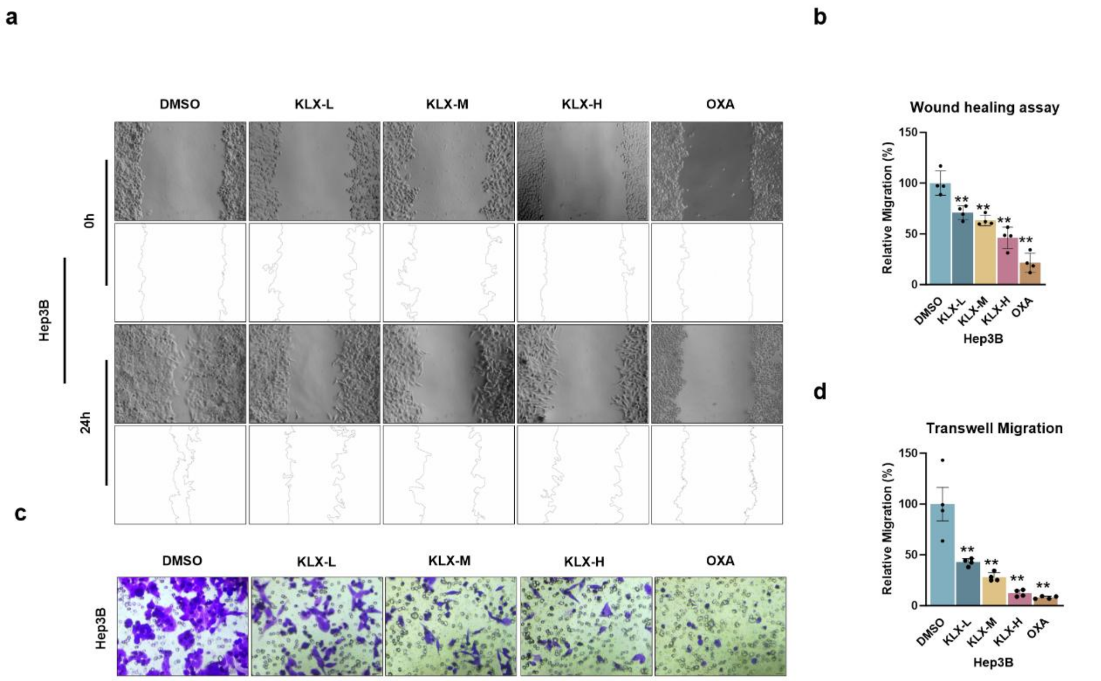

Fig. S2 The effect of KLX on Hep3B cell migration. **a, b** Wound healing assay results showing Hep3B cell migration after treatment with KLX or OXA, with images taken at 0 h and 24 h. *n*=4. **c, d** Transwell assays were also used to evaluate Hep3B cell migration. *n*=4. All the data are expressed as the means ± SDs; **P* < 0.05, ***P* < 0.01, vs. the DMSO group.

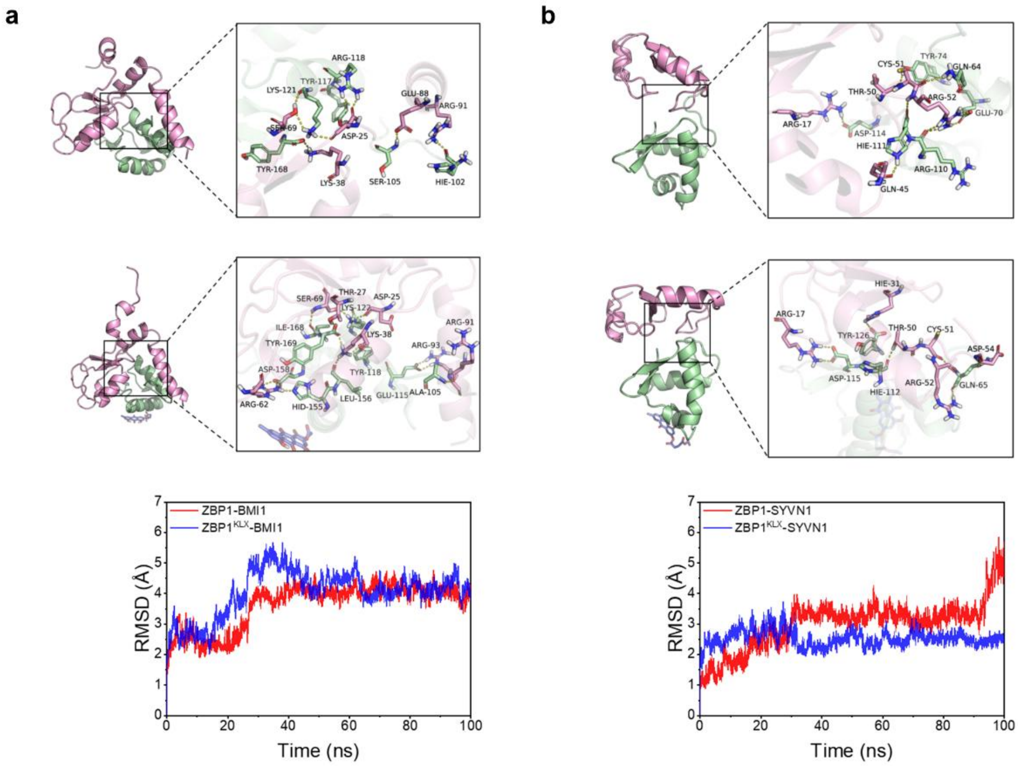

Fig. S3 Molecular dynamics simulation assessed the impact of KLX on the binding affinity between ZBP1 with BMI1 and SYVN1 proteins.

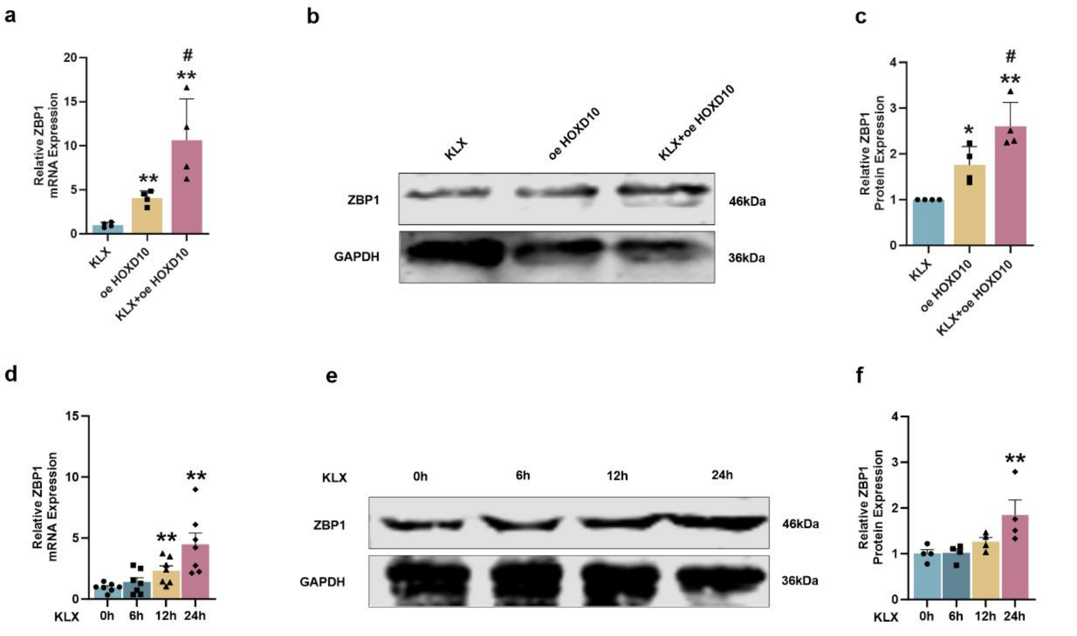

Fig. S4 The regulatory effects of KLX and HOXD10 on ZBP1. **a** qPCR experiment showing the effects of KLX and HOXD10 overexpression on ZBP1 mRNA levels. *n*=4. **b, c** Western blot analysis showing the effects of KLX and HOXD10 overexpression on ZBP1 protein levels. *n*=4. *$P < 0.05$, **$P < 0.01$, *vs*. KLX group. $^{\#}P < 0.05$, *vs*. oe HOXD10 group. **d-f** qRT-PCR and Western Blot experiments validated the effects of KLX on ZBP1 mRNA and protein expression at different time points. *n*=4-6.

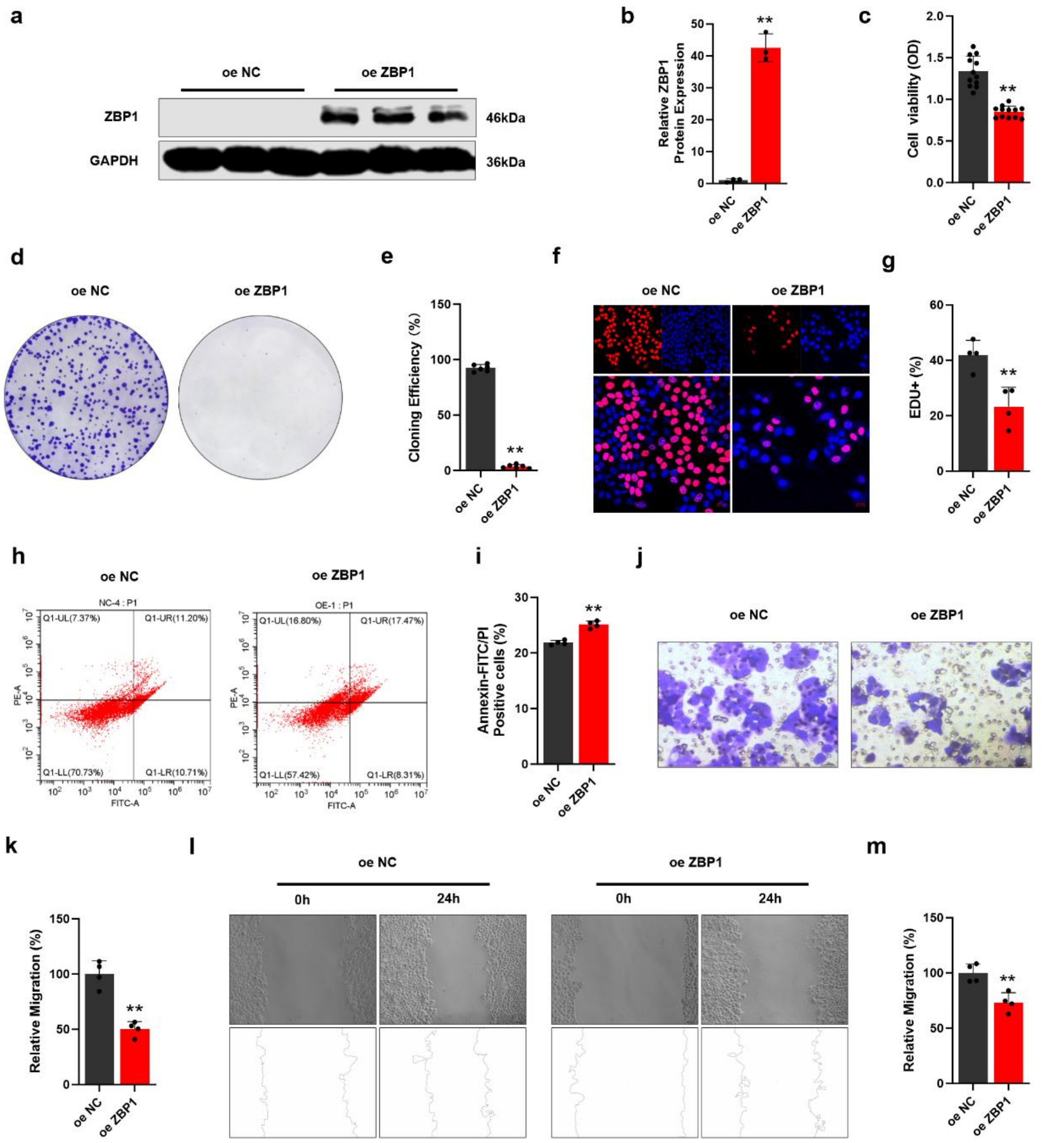

Fig. S5 Overexpression of ZBP1 inhibited the proliferation and migration of HepG2 cells. **a, b** Western blot analysis verified the efficiency of ZBP1 overexpression. **c** CCK8 assay confirmed that ZBP1 overexpression reduced HepG2 cell viability. **d-m** Colony formation, EdU, flow cytometry, Transwell assay and wound healing assay verified the effect of ZBP1 overexpression on HepG2 cells and the corresponding statistical graph. All data are expressed as mean ± SDs; *$P < 0.05$, **$P < 0.01$, *vs*. oe NC group.
